# The NAS Perchlorate Review: Questions Remain about the Perchlorate RfD

**DOI:** 10.1289/ehp.8254

**Published:** 2005-05-25

**Authors:** Gary Ginsberg, Deborah Rice

**Affiliations:** 1 Connecticut Department of Public Health, Hartford, Connecticut, USA; 2 Maine Bureau of Health, Augusta, Maine, USA

**Keywords:** NAS, neurodevelopmental, perchlorate, RfD, thyroid hormone

## Abstract

Human exposure to perchlorate is commonplace because it is a contaminant of drinking water, certain foods, and breast milk. The U.S. Environmental Protection Agency (EPA) conducted a perchlorate risk assessment in 2002 that yielded a reference dose (RfD) based on both the animal and human toxicology data. This assessment has been superceded by a recent National Academy of Science (NAS) review that derived a perchlorate RfD that is 20-fold greater (less stringent) than that derived by the U.S. EPA in 2002. The NAS-derived RfD was put on the U.S. EPA’s Integrated Risk Information System (IRIS) database very quickly and with no further public review. In this commentary we raise concerns about the NAS approach to RfD development in three areas of toxicity assessment: the dose that the NAS described as a no observable adverse-effect level is actually associated with perchlorate-induced effects; consideration of uncertainties was insufficient; and the NAS considered the inhibition of iodine uptake to be a nonadverse effect. We conclude that risk assessors should carefully evaluate whether the IRIS RfD is the most appropriate value for assessing perchlorate risk.

Perchlorate is a widespread contaminant in drinking water, food, and breast milk [California Department of Health Services (CDHS) 2005; Food and Drug Administration 2004; Kirk et al. 2005]. The U.S. Environmental Protection Agency (EPA) developed a draft risk assessment for perchlorate in 2002 (U.S. EPA 2002a, 2003), but this was recently superceded by an analysis by the National Academy of Science (NAS 2005). The NAS derived a perchlorate reference dose (RfD) that is approximately 20-fold higher (less stringent) than the U.S. EPA draft RfD from 2002. Without any further deliberation or public review, the U.S. EPA has adopted the NAS value and placed it on its Integrated Risk Information System (IRIS) website (U.S. EPA 2005), a primary source of data for state risk assessors.

Given the disparity between the initial U.S. EPA analysis (U.S. EPA 2002a) and the 2005 NAS report, the perchlorate RfD posted on IRIS (U.S. EPA 2005) merits careful consideration before health officials embrace this less stringent value. Our current purpose is to highlight issues with the primary human studies used in the NAS perchlorate determination. However, it is also worth noting that the NAS discounted the studies in rats, arguing that rats are more sensitive to the effects of perchlorate than are humans. We believe that the rat studies provide important information, particularly with respect to thyroid suppression, that should be considered in concert with the human data as part of a comprehensive risk assessment.

We present the outstanding toxicology issues, particularly with respect to the human studies, when considering the public health implications of perchlorate in drinking water and the diet.

## What Is the NOAEL?

A key step in deriving any RfD is finding a dose at which toxic effects can no longer be demonstrated—the no observable adverse effect level (NOAEL). This dose is believed to be below the threshold resulting in toxicity and so can be used to extrapolate a “safe” dose for the public, including sensitive subgroups. For perchlorate, there is disagreement over where this NOAEL exists. The critical study used by the NAS involved 14-day exposure of adult humans in which perchlorate induced a dose-dependent decline in iodine uptake into the thyroid (Greer et al. 2002). The U.S. EPA called the lowest dose in that study a LOAEL (lowest observable adverse effect level), whereas the NAS called it a NOAEL on the basis that, although there was a slight numerical difference from controls, it was not statistically significant. We should mention that the NAS actually used the term “NOEL” (no observed effect level), leaving out the “adverse” descriptor.

**Figure 1:**
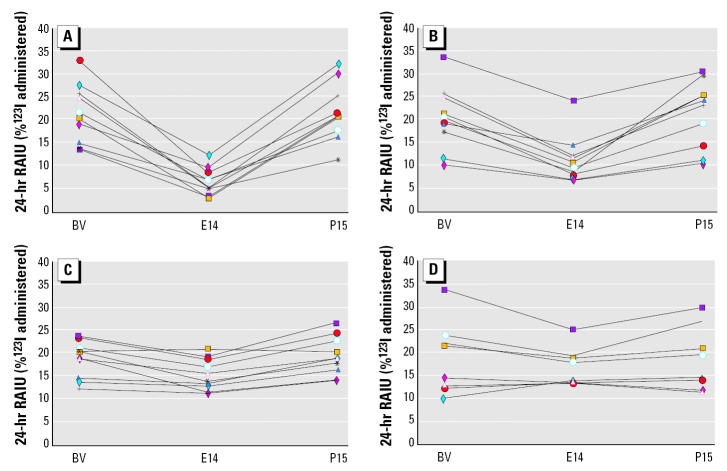
Radioiodine (^123^I) uptake (RAIU) inhibition profiles at various perchlorate doses in human subjects over 2 weeks of exposure, followed by 2 weeks of recovery. (*A*) 0.5 mg/kg/day. (*B*) 0.1 mg/kg/day. (*C*) 0.02 mg/kg/day. (*D*) 0.007 mg/kg/day. BV, baseline value. Reprinted from Greer et al. (2002) with permission from *Environmental Health Perspectives*.

Examination of Figure 1 (from Greer et al. 2002) reveals that in fact the low-dose group did show clear evidence of a perchlorate effect based on data from individual subjects. Of the seven subjects in the low-dose group (Figure 1D), three showed no perchlorate effect on radioiodine uptake. This is seen as the essentially flat line from baseline value through 2 weeks of perchlorate exposure (exposure day 14; E14) and 2 weeks of perchlorate-free recovery (postexposure day 15; P15). However, four low-dose subjects evidenced the characteristic perchlorate effect observed at the higher doses (Figure 1A–C). Their baseline values decreased after perchlorate exposure and returned to baseline thereafter. The subjects who were resistant to the perchlorate effect, as shown in Figure 1D, had baseline values for radioiodine uptake that were low (< 15%), whereas the responders all had values > 15%. This trend can be seen in the higher dose groups as well (Figures 1B,C), in which the greatest perchlorate effects are in those whose baseline uptake is highest. Baseline uptake may be high in those with induced levels of iodide transporter in response to suboptimal iodide intake (Dohan et al. 2003). How this may affect sensitivity to perchlorate is unclear, although it is possible that the ability of the method to detect perchlorate-induced inhibition in iodide uptake may be enhanced when starting at a higher baseline. Whatever the explanation, the individual results of Greer et al. (2002) point to an effect in four of the seven individuals tested at the lowest dose, indicating that this dose is an effect level.

The NAS, however, relied on an average of the group’s response, and called the low dose a NOAEL rather than a LOAEL. It is true that the group mean for the low dose was not statistically different from the control value, but this was due to the high degree of variability (Figure 1D). In fact, one “nonresponder” in the low-dose group had iodide uptake that was 140% of control. This is an influential data point that tends to obscure uptake inhibition in other subjects when data are pooled.

It is important to evaluate variable data sets to determine whether subgroups can be identified that may have a different threshold for an effect than the main group. This is especially true when evaluating human data because of the greater intersubject variability that can be anticipated relative to inbred animals. The data of Greer et al. (2002) point toward a more sensitive subgroup, which in this case represents more than half of the test group but is not evident in the group mean analysis due to the variable nature of the response. All of this points to the fact that the small number of individuals tested at each of the dose groups [*n* = 7 at the low dose (Greer et al. 2002)] and the variability inherent in the data resulted in weak power to identify statistically significant effects, even where closer inspection shows that they were present (U.S. EPA 2002a).

The influence of this determination on the RfD is direct: if the lowest dose is a LOAEL, then one estimates the NOAEL via application of an uncertainty factor (typically 3- or 10-fold) or via benchmark dose analysis. Either of these procedures would result in an RfD below that derived by the NAS (2005) and more consistent with that initially drafted by the U.S. EPA (2002a)

## How Much Uncertainty Is There in the Perchlorate Database?

Both the NAS (2005) and the U.S. EPA (2002a) applied a 10-fold intrahuman uncertainty factor, acknowledging that the critical study examined a small number of healthy, iodine-replete adults. Perchlorate effects on iodine uptake and thyroid hormone status may be more dramatic in pregnant women who are already somewhat iodine deficient, a high-risk scenario that is not uncommon (Azizi et al. 2003; Kibirige et al. 2004). Unlike the U.S. EPA’s 2002 assessment (U.S. EPA 2002a), the NAS’s assessment did not apply any other uncertainty factors. One dissenting member of the NAS committee thought that an additional uncertainty factor of 3-fold should be applied to account for additional issues.

We believe that a database uncertainty factor of 3- to 10-fold is warranted based upon key data gaps as follows.

### Potential for greater toxicity to newborns from lactational exposure.

Perchlorate is actively transported into breast milk, where relatively high levels have been reported in the United States and Chile (Gibbs 2004; Kirk et al. 2005). However, there is very little information on perchlorate effects from lactational exposure. This is an important data gap because perchlorate not only concentrates in breast milk but also inhibits iodide uptake into this medium (Kirk et al. 2005). Therefore, the nursing infant may receive less iodide at the same time that it receives a dose of perchlorate that may inhibit iodide use.

A rat study by Argus Research Laboratories, Inc. (2001), which involved a combination of gestational and lactational exposure, found postnatal effects on thyroid hormone status at low doses. However, it is difficult to separate the effects of lactational and gestational exposure in this study, and all doses were associated with effects, so a NOAEL was not established. Regarding human lactational exposure, there is also very limited information. The Chilean study (Crump et al. 2000) did involve lactational exposure to breast milk and found no effects on newborn thyroid-stimulating hormone (TSH) levels in areas with high levels of perchlorate in drinking water. However, women in the Chilean high-perchlorate area did not have lower iodine content in breast milk relative to the control area despite relatively high levels of perchlorate in breast milk (Crump et al. 2000; Gibbs 2004). This is in contrast to recent results in the United States in which there was an inverse relationship between perchlorate and iodide levels in breast milk (Kirk et al. 2005). The difference in breast milk iodide results may be related to the high intake of iodide in the Chilean areas studied, considerably higher than that common in the United States (NAS 2005). The postnatal parameters evaluated in the Chilean study (Crump et al. 2000) are also very limited (serum TSH concentrations, evidence of goiter). The lack of useful lactational exposure studies is a critical data gap that adds uncertainty to perchlorate risk assessment.

### Uncertain relationship between short-term and chronic perchlorate toxicity.

The key study is of 14 days’ duration (Greer et al. 2002). There is uncertainty that longer-term exposure may have a cumulative effect due to prolonged perturbation of iodine transport or increasing storage of perchlorate in the thyroid and other tissues. A study in rats suggests greater perchlorate toxicity to the thyroid from 90-day exposure than from 14-day exposure (Springborn Laboratories 1998). One longer-term study in humans was reported only as an abstract (Braverman et al. 2004). Three- or 6-month exposure to perchlorate caused no effects on thyroid hormone status or iodine uptake inhibition in small numbers of subjects (*n* ≤5/group). The dose levels should have been high enough to inhibit iodine uptake based on the 14-day studies of Greer et al. (2002). Although this might indicate an adaptive response to longer-term perchlorate exposure, this study (Braverman et al. 2004) lacked statistical analysis and has not been formally published or peer reviewed, and it is uncertain whether it had sufficient power to detect an effect.

## What Is an Adverse Effect?

Part of the NAS rationale for not including any other uncertainty factors is that they consider the critical end point, perchlorate-induced iodine uptake inhibition in the thyroid, as a precursor effect, but not an adverse effect (NAS 2005). The NAS states that perchlorate’s toxic effects on brain development would not occur unless thyroid hormone levels available to the fetus or neonate are diminished. Inhibition of iodine uptake is a step in this pathway, but compensatory mechanisms may prevent an effect on circulating thyroid hormone levels. The NAS stated that one would need a 75% inhibition in iodine uptake for the perchlorate effect to be adverse. However, this statement was not supported with evidence or references, and it is unclear whether the NAS meant this to apply to all individuals or just healthy adults replete in iodine and thyroid hormone.

The issue of what constitutes an adverse effect has been debated in risk assessment circles from time to time. Adaptive responses such as induction of hepatic metabolizing enzymes in response to agents such as phenobarbital are generally considered to be non-adverse and are not used to formulate an RfD (Williams and Iatropoulos 2002). However, precursor effects that are part of the toxic pathway are of concern. The risk assessment process acknowledges that other exposures or disease states may also affect the mode of action of the chemical under consideration or may contribute to the adverse effect by some other means. Regarding perchlorate, inhibition of iodine uptake may be compounded by factors that exist in at least some pregnant women and neonates—reduced stores of iodine and thyroid hormone. Therefore, the perchlorate precursor effect seen in healthy adult subjects should be viewed as a critical effect that warrants prevention.

This strategy is consistent with RfD-setting policy at the U.S. EPA (2002b), which states

The critical effect is defined as the first adverse effect, or its known precursor, that occurs to the most sensitive species, as the dose rate of the agent increases.

RfDs have been set based on precursor biochemical changes such as plasma or red cell cholinesterase inhibition for various organo-phosphates [e.g., chlorpyrifos (U.S. EPA 2004a) and malathion (U.S. EPA 2004b)].

Where the distinction between precursor and adverse effect may become important regards how large a LOAEL-to-NOAEL factor should be used. For mild or precursor effects, one might choose to use a smaller LOAEL-to-NOAEL factor than if one were extrapolating from a clearly toxic effect. For this reason, the LOAEL-to-NOAEL factor for perchlorate based upon iodine uptake inhibition (Greer et al. 2002) may reasonably be set at 3-fold rather than 10-fold. However, the distinction between precursor and adverse effect does not normally affect the size of other uncertainty factors considered in RfD determination, such as database deficiencies. Therefore, we do not see the distinction that the NAS made between precursor and adverse effect as affecting the RfD-setting process except as a consideration in choosing a LOAEL-to-NOAEL uncertainty factor.

The factors described above lead us to conclude that the perchlorate RfD derived by the NAS and now on IRIS (U.S. EPA 2005) is higher than what is needed to protect public health with a reasonable margin of safety. We recommend that risk assessors carefully evaluate the IRIS RfD in terms of whether this RfD is based upon a valid NOAEL and whether sufficient uncertainty factors have been applied. The importance of reevaluating the perchlorate RfD is underscored by the issues we raise in this commentary, by the potential for widespread human exposure *in utero* and for nursing infants, and because there has been no opportunity for public comment on the U.S. EPA’s IRIS value (U.S. EPA 2005) once the NAS completed its review (NAS 2005).
